# Recent Trends in Antimicrobial Resistance among Anaerobic Clinical Isolates

**DOI:** 10.3390/microorganisms11061474

**Published:** 2023-06-01

**Authors:** Sophie Reissier, Malo Penven, François Guérin, Vincent Cattoir

**Affiliations:** 1 Rennes University Hospital, Department of Clinical Microbiology, F-35033 Rennes, France; sophie.reissier@chu-rennes.fr (S.R.); malo.penven@chu-rennes.fr (M.P.); francois.guerin@chu-rennes.fr (F.G.); 2 UMR_S1230 BRM, Inserm, University of Rennes, F-35043 Rennes, France; 3 CHU de Rennes, Service de Bactériologie-Hygiène Hospitalière, 2 Rue Henri Le Guilloux, CEDEX 9, F-35033 Rennes, France

**Keywords:** anaerobes, antimicrobial resistance, evolution, metronidazole, clindamycin, β-lactams

## Abstract

Anaerobic bacteria are normal inhabitants of the human commensal microbiota and play an important role in various human infections. Tedious and time-consuming, antibiotic susceptibility testing is not routinely performed in all clinical microbiology laboratories, despite the increase in antibiotic resistance among clinically relevant anaerobes since the 1990s. β-lactam and metronidazole are the key molecules in the management of anaerobic infections, to the detriment of clindamycin. β-lactam resistance is usually mediated by the production of β-lactamases. Metronidazole resistance remains uncommon, complex, and not fully elucidated, while metronidazole inactivation appears to be a key mechanism. The use of clindamycin, a broad-spectrum anti-anaerobic agent, is becoming problematic due to the increase in resistance rate in all anaerobic bacteria, mainly mediated by Erm-type rRNA methylases. Second-line anti-anaerobes are fluoroquinolones, tetracyclines, chloramphenicol, and linezolid. This review aims to describe the up-to-date evolution of antibiotic resistance, give an overview, and understand the main mechanisms of resistance in a wide range of anaerobes.

## 1. Introduction

Anaerobes are well known to be an important part of the normal human intestinal, vaginal, oral, and skin microbiota [1]. Anaerobic bacteria are also opportunistic pathogens that could be involved in various types of human infections in association with aerobic bacteria, such as brain abscesses, intraabdominal, skin, or pelvic infections. They can also cause monomicrobial infections such as bacteremia, deep tissue infections, and bone and joint infections. These infections are associated with severe morbidity and a high rate of mortality [2,3]. In addition to the well-known anaerobes such as *Bacteroides* spp. or *Clostridium perfringens*, new genera and species are regularly described through improvements in culture and identification techniques and implicated in severe human infections. Clinically relevant Gram-negative anaerobic bacteria include *Bacteroides fragilis* group, *Prevotella* spp., *Fusobacterium* spp., and *Veillonella* spp. [4]. The main Gram-positive bacilli isolated from clinical samples are *Cutibacterium* spp., especially *C. acnes* (formerly known as *Propionibacterium acnes)*, which is involved in chronic bone and joint infections. Additionally, *Clostridium* spp. (apart from *Clostridioides difficile*) are responsible for many types of severe infections, such as gas gangrene. *Actinomyces* spp., isolated in deep tissue infections, is also noteworthy [5,6,7]. *Finegoldia magna* and *Parvimonas micra* are the two most isolated Gram-positive anaerobic cocci (GPAC).

Due to technical and financial constraints associated with the identification and culture of anaerobic bacteria, microbiology and antibiotic susceptibility testing (AST) of anaerobes isolates are rarely routinely performed in clinical microbiology laboratories [1]. Therefore, the treatment of anaerobic infections has long been empirical, which has led to therapeutic failures and the emergence of resistance [8]. Over the last two decades, a growing number of studies around the world have focused on describing the epidemiology of resistance in anaerobic bacteria, with a worldwide increase despite differences between countries [9]. However, AST has been performed differently in laboratories according to countries, most of the time without following CLSI and EUCAST methods [4]. For example, Asian laboratories mostly used micro-dilution-based techniques, while the majority of laboratories in Europe and the US employed gradient diffusion strips or agar dilution, which is the reference method. Breakpoints for AST interpretation also sometimes differ between EUCAST and CLSI guidelines. This diversity of practices could lead to great variability in results and make studies difficult to compare. Moreover, there is a scarcity of recent comprehensive epidemiological data with a significant number of strains. 

The aim of our study was to describe the evolution of resistance among anaerobic clinical isolates and summarize the mechanisms of resistance to the main antibiotics used in the treatment of anaerobic infections. To analyze the evolution of resistance epidemiology, articles published on PubMed between 2013 and 2023 were selected. Specifically, we included only those articles in which the authors used MALDI-TOF mass spectrometry or sequencing to identify anaerobes. This review only focused on clinical isolates, whereas data about anaerobes isolated from healthy people wAS not included. We also excluded results concerning *C. difficile* as the therapeutic management of infections caused by this pathogen is species-specific.

## 2. β-Lactams

β-lactam antibiotics are considered the drugs of choice in the management of anaerobic infections. This is due to their broad spectrum of activity, low toxicity, and continued efficacy against almost all anaerobic species, especially when used in combination with β-lactam/β-lactamase inhibitors (BL/BLI) or carbapenems. Among the anaerobes, *Bacteroides* and *Parabacteroides* species are of greatest concern considering their higher resistance rates. In the 1990s, in Europe, a large multicenter study in 15 different countries reported a prevalence of 1%, 3%, and 0.3% for amoxicillin/clavulanate (AMC), cefoxitin, and imipenem among the *B. fragilis* group (n = 1289) [10]. Over the past 20 years, nearly 10% have become resistant to AMC and piperacillin/tazobactam (PTZ), while 17% and 1% are resistant to cefoxitin and carbapenems, respectively [11]. In Canada, an increase in resistance to AMC was also observed between 1992 and 2010–2011 (from 0.8% to 6.2%), while a slight decrease in cefoxitin resistance was reported (26% vs. 15%), potentially related to reduced use of cefoxitin [12]. In the US, an increase in the resistance rate of ampicillin/sulbactam (from 4% to 6%) and PTZ (from 2% to 7%) was observed among *Bacteroides* and *Parabacteroides* isolates between 2007–2009 and 2010–2012 [13,14]. An increase in carbapenem resistance was also reported, such as in Poland, where imipenem resistance increased between 2007–2012 and 2013–2017 in the *Bacteroides fragilis* group (0.5% to 2.2%), especially in non-*fragilis Bacteroides* (1.4% to 3.7%) [15]. A decrease in susceptibility to meropenem among the *B. fragilis* group was also reported in Japan between 2010 and 2018–2019 (98% to 90%) [16]. In recent studies, AMC resistance ranges from 2 to 9% in *B. fragilis,* except in Spain, where higher rates were reported (29%) [15,17,18,19,20]. PTZ resistance remains lower, with a resistance rate varying between 1 and 3%, while the resistance rate reaches 5% in Korea and Greece [17,21,22,23]. Higher AMC and PTZ resistance rates were reported in the *B. fragilis* group excluding *B. fragilis*, especially in *B. thetaiotaomicron* and *Phocaeicola vulgatus* [17,18,23]. In *B. fragilis*, the rate of resistance to carbapenems ranges from 0 to 5% for imipenem and from 2 to 5% for meropenem [15,17,18,19,20,21,23,24]. 

Among *Prevotella* spp., a slight increase in penicillin-resistant isolates was described in Belgium between 1993–1994 (52%) and 2011–2012 (65%), while a higher increase was observed in Bulgaria between 2003–2004 (15%) and 2007–2009 (61%) [13,25]. In recent studies, most isolates are resistant to penicillin, with a prevalence of 60–80% in European countries, except for Germany, where Wolf et al. noted a lower rate (36%) [13,19,26,27]. Over the world, resistance rates were similar in the US (65%) and Canada (63.5%), while a higher resistance rate was noted in Korea (91%) [21,22,28]. However, most strains remain susceptible to BL/BLI combinations and carbapenems, except in Canada and Spain, where a few strains resistant to PTZ and AMC were reported [19,22]. Among *Fusobacterium* spp., the rate of resistance to penicillin range between 5 and 17%, while a higher prevalence was reported in Ireland (50%) [17,19,21,22,27]. BL/BLI combination and carbapenems still have excellent activity and only some resistant isolates have been sporadically reported [17,19,22]. *Veillonella* spp. have high rates of penicillin resistance, ranging from 29 to 55%, except in Korea, where Buyn et al. reported 100% of resistance (n = 11), in contrast to Ali et al., who reported no resistant strains in Ireland (n = 9) [17,19,21,22,27,28]. Among *Veillonella* spp., high levels of resistance to TZP (MIC ≥ 128 mg/L) were observed [17,21,22]. 

In Gram-positive anaerobic bacteria, most isolates of *Propionibacterium* spp., *Cutibacterium* spp., *Finegoldia magna*, *Peptoniphilus* spp., *Anaerococcus* spp., and *Parvimonas micra* are susceptible to β-lactams [18,19,22,27,28,29]. Penicillin resistance in *Peptostreptococcus anaerobius* appears to be more common and ranges from 5 to 25%, although a higher rate of resistance to ampicillin has been reported in France by Guérin et al. (55%, 5/11) [29,30,31]. In *Clostridium* spp., penicillin resistance is higher, varying between 11–30% worldwide, and only a few strains are resistant to BL/BLI combinations and carbapenems. *C. perfringens* exhibits a lower resistance rate, ranging from 0 to 5% [13,21,22,22,27,28,28,30,31]. In *Eggerthella lenta*, resistance to penicillin was commonly recovered (13–98%), while low susceptibility levels have been observed for TZP, with MIC_50_ ranging between 16 and 32 mg/L [21,22,32]. The ranges of MIC, MIC_50,_ and MIC_90_ are synthetized for AMC (Gram-negative) and penicillin (Gram-positive) in Table 1 and Table 2.

Mechanistically, β-lactam resistance occurs by three different mechanisms: enzymatic inactivation, target modification, and decreased intracellular concentration by active efflux and/or porin alteration. In *B. fragilis*, three β-lactamases, namely CepA, CfxA, and CfiA, drive β-lactam resistance by enzymatic inactivation. CepA and CfxA, belonging to functional subgroup 2e cephalosporinase, hydrolyze penicillins and cephalosporins (except cephamycins for CepA). CepA coding by *cepA*, a chromosomal cephalosporinase recovered in 70–90% of isolates, did not always correlate with antibiotic resistance. The gene *cepA* remains often low-levelly expressed, with over-expression occurring by IS insertion upstream [24,33,34,35]. In *B. fragilis*, cfxA is less common, recovered among 3–20% of *B. fragilis,* but more often associated with phenotypic β-lactam resistance. The gene *cfxA* is carried mainly by the mobilizable transposon MTn*4555* that integrates the IS upstream gene, which promotes high-level expression [24,33,34,36,37]. The gene *cfxA* is more common in non-*fragilis Bacteroides,* while *cepA* carriage is occasionally recovered [24,33,38]. 

Carbapenem resistance in *B. fragilis* is primarily promoted by the class-B metallo-carbapenemase CfiA, encoded by a chromosomal gene recovered in some strains. In terms of intra-species diversity, *B. fragilis* can be classified into two subgroups based on the presence or absence of the *cfiA* and *cepA* genes. These subgroups are referred to as division I (*cfiA*-) and division II (*cfiA*+). Subgroup division is achieved by DNA–DNA hybridization and ribotyping, while detection is now available by matrix-assisted laser desorption/ionization time-of-flight mass spectroscopy [39,40,41]. In a retrospective study, Ferløv-Schwensen et al. observed an increase in division II bacteroides among clinical isolates (2.8% vs. 7.8%) between 1973–1991 and 2002–2015, potentially due to the overuse of carbapenems [42]. Recent studies have reported a similar prevalence of *cfiA* in Europe, ranging between 8 and 16% [24,34,43]. In Asia, close rates have been reported, with a prevalence of 15% and 22% in Japan and China, respectively [33,44]. Curiously, a higher prevalence of division II (*cfiA*+) has been reported in bloodstream infections compared to other clinical isolates [43]. The gene *cfiA* is not always correlated with phoneticphenotypic resistance related to low-level expression. Insertion sequences upstream of the gene, mainly belonging to the IS1*380* family, are the cornerstone to induce their overexpression, which leads to phenotypic resistance to β-lactams [38,45,46]. It should be noted that the use of meropenem (not imipenem) +/− EDTA allows the detection of *cfiA*+ strains with low-level expression [47]. In the non-*fragilis Bacteroides* group, only *cfxA* is generally recovered, and an unknown mechanism provides resistance to β-lactams in non-*cfxA* isolates [34]. However, in recent studies, Soki et al. identified in *B. xylanisolvens*, a non-fragilis *Bacteroides* species, the *crxA* gene coding for a metallo-B-carbapenemase close to *cfiA* that confers resistance to carbapenem, while Wallace et al. identified putative class A β-lactamases among non-fragilis *Bacteroides* [45,48]. In *Prevotella* spp., *cfxA* variants (*cfxA2*, *cfxA3*, *cfxA6,* and *cfxA7*) are associated with ampicillin resistance, with a prevalence ranging between 51–78% [34,49,50,51]. The *cfxA2* gene differs from the *cfxA* of *P. vulgatus* by an amino acid change, while *cfxA3*, *cfxA6,* and *cfxA7* differ by two [49]. 

β-lactamases in other anaerobes are less studied, but penicillinases are outlined, mainly by phenotypic approaches in *Fusobacterium* spp., *Porphyromonas* spp., and *Clostridium* spp. [52,53,54,55]. Target modification by alteration of penicillin-binding proteins (PBPs) promotes cefoxitin resistance in the *Bacteroides fragilis* group. In fact, the modification of PBPA or PBP3 appears to play a greater role than hydrolysis by CfxA [37,56,57]. A decrease in imipenem susceptibility was also associated with PBP2Bfr modifications [58]. In *C. perfringens*, PBP alterations following β-lactams exposure result in decreased affinity of Β-lactams for PBP1 but an overproduction of PBP6 related to phenotypic resistance to penicillin G and ceftriaxone [59,60]. 

In *Veillonella* spp., PBP modification leads to high-level resistance to TZP (MIC >128 mg/L) in β-lactamase-negative isolates, whereas ampicillin remains active (MIC = 0.5–4 mg/L) due to a retained affinity for PBP [61]. In *B. fragilis*, derepression *of bmeABC* coding for a RND efflux pump can trigger the extrusion of ampicillin, cefoperazone, and cefoxitin [62]. Resistance induced by porin loss remains poorly studied, while in *B. thetaiotaomicron*, it has been suggested that resistance to AMC may be related to a defect in the expression or absence of a porin [63]. Moreover, loss of porin associated with PBP alteration may be co-induced following cefoxitin exposure leading to ampicillin and cephalosporin resistance in *B. thetaiotaomicron* [57].

## 3. Metronidazole

Metronidazole, a drug of the 5-nitroimmidazole family, is an old molecule introduced in the 1960s but is still one of the most important antibiotics for the management of anaerobic infections. Mechanistically, metronidazole is a prodrug that remains inactive until absorbed by passive diffusion and activated intracellularly. The reduction in the nitro group leads to the formation of a toxic metabolite that interacts with several macromolecules in the cell, mainly DNA, leading to strand breaks and cell death. Enzymatic reduction is principally driven by pyruvate ferredoxin/flavodoxin oxydoreductase (PFOR) and occurs only at low oxygen concentrations [64,65]. In 1978, the first clinical isolate of *B. fragilis* selected by long-term treatment with metronidazole was described [66]. Currently, the prevalence of resistance to metronidazole is relatively low in Gram-negative bacilli, with higher proportions in *Bacteroides* spp., *Parabacteroides* spp. (<3%), and *Prevotella* spp. (<5%) [13,15,17,18,19,20,21,22,23,24,27,28]. Some countries reported a higher prevalence of resistance in *Prevotella* spp., such as Germany and Spain, where 12% (3/25) and 7% (2/30) of isolates were resistant, respectively [26,30]. In other species, resistance is globally lower, but higher resistance rates have been sporadically reported in small studies in *Veillonella* spp. (23–27%, 3/14 and 3/11), *Peptoniphilus* spp. (13%, 3/21), *Anaerococcus* spp. (13%, 4/31), *P. micra* (9–12%, 1/11 and 2/17), and *Clostridium* spp. (8%, 3/37) [19,21,30,31]. Some resistant isolates of *Phorphyromonas* spp. and *Alistipes* spp. have also been reported [26]. Gram-positive bacteria such as *Actinomyces* spp., *Cutibacterium* spp., and *Propionibacterium* spp. are intrinsically resistant to metronidazole. This is probably due to the absence of the PFOR system or an alternative pathway of pyruvate catabolism [67]. However, susceptible isolates have rarely been reported in this species (<5%), while higher susceptible rates were observed in the US for *Actinomyces* spp. (16.2%) [13,18,22,28]. Over the past years, resistance to metronidazole appears to be stable and rare, which allows this old antibiotic to maintain a key role in the management of infections caused by anaerobes [13,25]. However, it remains difficult to evaluate resistance evolution worldwide considering the different clinical breakpoints of CLSI (≥32 mg/L) and EUCAST (>4 mg/L). The ranges of MIC, MIC_50_, and MIC_90_ are synthetized in Table 3 and Table 4.

Resistance to metronidazole occurs through a complex mechanism mediated by decreased drug activation, limited absorption, or increased DNA repair. One of the most important and well-studied mechanisms is the pro-drug inactivation via nitro-imidazole reductase coding by *nim* genes that transform metronidazole into a non-toxic compound. Up to now, 12 *nim* genes (*nimA* to *nimL*) sharing > 50% nucleotide similarity have been detected worldwide, mainly in *Bacteroides* spp. and *Prevotella* spp. [67,69]. Few studies focused on the prevalence of *nim* genes, and 2–3% of *Bacteroides* spp. carry them with a wide diversity (*nimA–H*, *nim J*, and *nimL*) [13,70]. Higher rates have been reported recently in India, where 61% of isolates were positive for *nim* genes (34/56) [71]. In *Prevotella* spp., the prevalence ranges from 3.7 to 5.3% (*nimA–C*, *nimI,* and *nim K*) [67,72,73]. In other Gram-negative anaerobes, *nim* genes have been reported in *Fusobacterium* spp. (*nimD*), *Porphyromonas* spp. (*nimC*), and *Veillonella* spp. (*nimE*) [67,73,74]. In Gram-positive bacteria, *nimB* carriage was reported in *Peptostreptococcus* spp., *F. magna*, *Anaerococcus prevotti,* and *P. micra* [75]. These resistance genes may be chromosomally located or harbored by plasmids, which may raise concerns about the spread of these resistance genes among anaerobic bacteria [4,67]. The *nim* genes are not always correlated with phenotypic antibiotic resistance (MIC = 1–6 mg/L), which is related to the low-level expression [76]. However, it is well recognized that high levels of resistance can be conferred by these “silent” genes, especially after metronidazole exposure, and insertion sequence (IS) is the cornerstone of their over-expression [76,77]. Other resistance mechanisms have been described, but their relevance in clinical isolates remains difficult to estimate. Metronidazole resistance in *Bacteroides* spp. can be related to complex metabolic changes in metabolism, such as a decrease in PFOR activity associated with an overproduction of LDH activity, an increase in rhamnose catabolism, or a modification of iron transport [78,79,80,81]. Another way to escape the antibiotic effect is the metronidazole extrusion by BmeABC, a RND family efflux transporter of *Bacteroides* spp. [62]. Overexpression of the DNA repair system RecA in *B. fragilis* can overcome DNA damage caused by metronidazole [82].

## 4. Clindamycin

Clindamycin is a bacteriostatic lincosamide active against Gram-positive cocci and anaerobes. This antibiotic is widely used in many countries due to its low cost, good tolerability, and the existence of susceptibility-testing recommendations. Because of its bacteriostatic activity, clindamycin is not recommended in the treatment of severe infections, for which bactericidal antibiotics such as β-lactams or metronidazole are preferred. Clindamycin, due to its broad spectrum of activity against anaerobes, has traditionally been used as a standard treatment for non-severe anaerobic infections. However, over the past 20 years, numerous cases of resistance have emerged, leading to clindamycin being one of the antibiotics with the highest rates of resistance among all anaerobic bacterial species [4]. 

In the 1990s, the prevalence of clindamycin resistance in *Bacteroides*/*Parabacteroides* spp. was less than 10% in European countries, the US, and Canada. However, over a span of 20 years, this resistance has more than tripled, reaching a prevalence of 35% in the US, Canada, and Europe [11,12,13,83,84]. Studies with more recent data confirmed that the rates of clindamycin resistance remained high and variable from one region to another. This variability is even more important as not all authors used the same AST techniques and breakpoint values. Indeed, CLSI’s recommendations differ from those of EUCAST.. In recent studies that considered all *Bacteroides* and *Parabacteroides* species, the resistance rate remained a concern and reached 33.5% in Italy, 49% in Spain and France, and 80% in Korea [21,30,85,86]. Curiously, clindamycin resistance was much lower in Scandinavian countries and Ireland; authors described a prevalence of around 17% [27,87,88]. For *B. fragilis*, the resistance rate was between 20 and 40% in European and North American countries and 50% in Asian countries, but there were many differences between countries [17,18,19,22,28,89]. In Japan, Ueda et al. described a significant increase in clindamycin resistance between 2010 (40.7%) and 2018–2019 (61.1%) [16]. In contrast, Kierkowska et al. showed that clindamycin resistance in Poland slightly decreased, from 22.5% to 18.3% between the 2007–2012 and 2003–2017 periods [15]. For other *Bacteroides* species belonging to the *fragilis* group, all studies described a higher rate of resistance (44% in the US, more than 50% in Canada, European, and Asian countries) [16,17,18,19,21,22,23,28,33]. The prevalence of clindamycin resistance was also important in *Prevotella* spp. strains, regardless of the country considered. It was around 30–40% in European countries and in the US, except in Ireland, where Ali et al. reported a higher rate (66.9%), and in Germany, where Wolf et al. reported a lower rate (24%) [17,19,26,27,28,30,89]. In Asia, the resistance rate was also significant (45% in Korea and 62.1% in Japan) [21,23]. *Fusobacterium* spp., *Veillonella* spp., and *Porphyromonas* spp. are also involved in anaerobic infections and are typically susceptible to clindamycin. Clindamycin resistance rates varied according to the regions, ranging from 6.7 to 25% and from 0 to 17% for *Fusobacterium* spp. and *Veillonella* spp., respectively [17,19,21,22,23,26,27,28,30,68]. In other Gram-negative anaerobes, such as *Porphyromonas* spp. or *Alistipes* spp., clindamycin resistance was rarely described [23,26,27]. 

Studies about antibiotic resistance in Gram-positive anaerobes are fewer in number. In European and North American studies on *Cutibacterium* spp. involved in infectious diseases, the authors reported a low prevalence of clindamycin resistance. Indeed, the resistance rate was about 8% or less in Spain, Italy, Ireland, and Austria, and around 10% in the US and Canada [18,19,22,27,28,30]. In Japan, Koizumi et al. described variable resistance rates according to species. *Cutibacterium avidum* was the most resistant species, with a resistance rate of 25%, which is consistent with other studies on *Cutibacterium* species [90,91]. As *Actinomyces* spp. is difficult to cultivate, AST is rarely performed in routine microbiology laboratories, and few studies on antibiotic resistance are available. Nevertheless, clindamycin resistance now appears to be substantial in most of the studies published. Except for Ireland, where Ali et al. described a resistance rate of 7.5%, resistance rates ranged from 18 to 44% in Europe and North America [18,22,27,28,30,31,92]. For *Clostridium* species other than *C. difficile*, as for other bacteria, the susceptibility to clindamycin was variable according to the country and AST techniques. Globally, most of the studies described a moderate activity of clindamycin against *Clostridium* spp., with resistance rates ranging from 16 to 30% in the US, Europe, and Korea [19,21,22,28,31,68]. Two European studies found lower resistance rates. In Ireland, Ali et al. observed 8.4% of resistant strains, similar to Sarvari et al. in Hungary [7,27]. Of all the non-*difficile Clostridium* species, *C. perfringens* is the most frequently isolated. Clindamycin resistance appeared to be lower than in other *Clostridium* species. In the US and Europe, authors described 10% or less of resistant strains, except in Greece, where Maraki et al. found a resistance rate of 40% [14,19,28,31,68]. Forbes et al. published a resistance rate of 14.6% in Canada. Limited studies have specifically investigated GPAC isolates, with the majority of them presenting data on GPAC as a whole without differentiating between specific species. However, it is worth noting that susceptibility to clindamycin appears to exhibit heterogeneity among GPAC species [29]. Except in Korea, clindamycin has limited activity against *F. magna*. Resistance rates were around 25% in the US, Spain, France, and Italy; around 35% in Canada; and above 50% in Greece [19,21,22,28,29,31,68]. On the other hand, a similar trend was described by the same authors for *Peptoniphilus* spp., *P. micra,* and *P. anaerobius*, which appeared to be more susceptible to clindamycin with resistance rates of 10% or less [22,28,29,30,31,68]. The ranges of MIC, MIC_50,_ and MIC_90_ are synthetized in Table 5 and Table 6.

Resistance to macrolides, lincosamides, and streptogramins (MLS) in Gram-negative anaerobes is mainly mediated by rRNA methylases encoded by *erm* genes (Table 7). In *Bacteroides* group and *Prevotella* spp., the majority of clindamycin-resistant strains harbored *erm*(F) gene [33,88]. Both *erm*(G) and *erm*(B) were also identified in some resistant isolates [70]. It is important to note that the presence of *erm* genes is not systematically correlated with phenotypic resistance to clindamycin. Indeed, Eitel et al. and Hashimoto et al. found that the prevalence of *erm*(F) was not significantly different in clindamycin-susceptible or clindamycin-resistant *Bacteroides* isolates. This implies that the expression of several resistance genes is necessary to observe phenotypic resistance [24,33]. Some other genes, *lin*(A), *mef*(A), and *msrSA*, may also be responsible for MLS resistance in *Bacteroides*, but they are rarely searched compared to *erm* genes. The *lin*(A) gene is located on a transposon (NBU2) and encodes for an O-nucleotidyltransferase, responsible for clindamycin and lincomycin resistance [24,93]. The *msrSA* and *mef*(A) genes are not specific to *Bacteroides* and mediate efflux-pump mechanisms [24]. Limited data are available on resistance mechanisms in Gram-positive anaerobes. As in Gram-negative anaerobes, clindamycin resistance seems to be mainly mediated by *erm* genes but data differed from one study to another. Koieumi et al. have shown that *erm*(X) is more prevalent than 23S rRNA mutations in *Cutibacterium* clinical isolates, especially in *C. avidum* [90]. In contrast, *erm*(X) was not detected in any resistant strain in the Oprica et al. study, suggesting that several mechanisms are involved [94]. Little is known about the genetic basis of resistance to MLS in GPAC. Only three studies have been published on clindamycin resistance mechanisms, and all three linked the resistance to erm gene expression, specifically *erm*(A), subclass *erm*(TR), and *erm*(B) [29,95,96]. Other mechanisms are probably involved, and more studies are needed to understand MLS resistance in Gram-positive anaerobes.

## 5. Fluoroquinolones

The class of fluoroquinolones includes many molecules, but due to their difficulties in reaching target sites, they are usually considered ineffective against anaerobes [4]. Moxifloxacin is currently the only antibiotic that demonstrates activity against anaerobic bacteria. However, since its approval by the FDA, numerous species have developed resistance mechanisms against this drug. In their review, Boyanova et al. observed a worldwide significant increase in resistance to moxifloxacin between 2000 and 2010, in *Bacteroides*/*Parabacteroides* spp. and *Clostridium* spp. [11,13,25,84,97]. In 2017, Gajdacs et al. reported similar results [4]. This upward trend has since been confirmed. In recent studies about *Bacteroides*/*Parabacteroides* spp., the prevalence of moxifloxacin resistance was between 30 and 42% [21,30,85]. In Asian and European countries, resistant strains represented about 20% and 30% of *B. fragilis* and non-*fragilis Bacteroides* isolates, respectively [16,17,21,23,24]. In the US, Hastey et al. observed higher resistance rates (26% for *B. fragilis* and 30% for the *B. fragilis* group without *B. fragilis*) [14]. Resistance was also significant in other Gram-negative anaerobes such as *Fusobacterium* spp. and *Prevotella* spp. However, the results of different studies varied considerably, with resistance rates ranging from 7 to 50% for *Fusobacterium* spp. and from 9 to 88% for *Prevotella* spp. It is important to note that, as for other antibiotics, AST was performed differently according to laboratories. Additionally, breakpoint values for AST interpretation differed between EUCAST and CLSI guidelines, making interpretation and comparison of studies difficult [98]. Indeed, a recent Italian study using the EUCAST criteria showed that 81% of *Bacteroides* spp. strains were resistant to moxifloxacin, whereas using the CLSI criteria, only 40% of these same strains were categorized as resistant [99]. Moreover, the authors used the agar dilution method to perform AST, which is recommended by EUCAST and CLSI guidelines and observed the highest resistance rates. This suggests that resistance rates determined by E-test or microdilution may be underestimated. Nevertheless, the prevalence of moxifloxacin resistance in anaerobes appears to be increasing worldwide.

Resistance to moxifloxacin in the *B. fragilis* group mainly resulted from chromosomal mutations (*gyrA*, *parC*) leading to target modification or active efflux [4,8,100]. Some studies have suggested that the multidrug efflux pump BexA could also be involved in moxifloxacin resistance in *B. fragilis*, but it remains unclear. Indeed, in Eitel et al. and Rong et al. studies, *bexA* was detected in some moxifloxacin-susceptible strains but not constantly resistant strains [24,38]. In *B. thetaiotaomicron*, *bexA* is overexpressed and confers constitutive low-level resistance to fluoroquinolones [101]. Resistance mechanisms in other anaerobic bacteria have been little explored until now.

## 6. Chloramphenicol

Chloramphenicol is a bacteriostatic antibiotic with good in vitro activity against most anaerobic bacteria. It has long been used in severe anaerobic and central nervous system (CNS) infections because of its lipid solubility and excellent diffusion in the CNS [102]. Currently, chloramphenicol is rarely used because of its toxicity. Side effects associated with chloramphenicol, particularly hematological complications but also cardiac and neurological issues, are infrequent but can be severe. Hematological side effects include bone marrow suppression and hemolytic anemia, which can have serious consequences. Additionally, rare cases of fatal aplastic anemia have been reported, and these effects are often irreversible [103]. Due to its limited use, chloramphenicol susceptibility is rarely tested routinely and few data are published. In vitro chloramphenicol resistance seemed to be exceptional and mainly described in Bacteroides spp. strains, even with breakpoint values that differed between EUCAST and CLSI guidelines [17,19,21,23,31,86]. Some therapeutic failures have been reported in patients with anaerobic sepsis treated with chloramphenicol, suggesting that in vitro susceptibility may not be correlated with clinical efficacy [104]. In *Bacteroides* spp., resistance to chloramphenicol is plasmid-mediated by the gene *cat*, which codes for an acetyltransferase that inactivates the drug [4,103,104].

## 7. Tetracyclines

Tetracycline is a bacteriostatic broad-spectrum antibiotic. Although it was initially active on anaerobes, this molecule now has limited usefulness due to the development of resistance in most anaerobic bacteria. These resistances were described a long time ago [105]. Some recent studies also observed high resistance rates in most anaerobes, although tetracycline susceptibility is rarely tested routinely [4,21,23]. Minocycline and doxycycline are second-generation tetracyclines that appeared to be more active than tetracycline, but as AST was not always performed in laboratories, few data are available and some resistance is described [90,106,107]. Since many resistance mechanisms affecting tetracycline can also affect minocycline and doxycycline, it is advisable to perform AST before using them. However, CLSI and EUCAST do not give clinical breakpoints for these drugs since there is no correlation between MIC and clinical outcome. This makes the use of these molecules even more difficult. Tigecycline is a third-generation tetracycline active against Gram-positive and Gram-negative anaerobes and approved to treat complicated infections, including those due to anaerobes [108,109,110]. As for minocycline and doxycycline, there are no breakpoint values available for MIC interpretation. Several studies described low tigecycline MICs for Gram-positive and Gram-negative anaerobes, suggesting an absence or a low level of resistance [17,26,31,89]. However, some resistant *B. fragilis* strains have been observed [24,83,85]. 

Tetracycline resistance involves three types of mechanisms in anaerobes: the efflux pump, ribosomal protection or modification, and enzymatic degradation, most of which are mediated by *tet* genes. More than 20 tetracycline-resistant efflux genes are identified, but only *tet*(A) to *tet*(E) and *tet*(K)-(L), which confer resistance to tetracycline and doxycycline, have been so far described in anaerobes, mainly in the *B. fragilis* group [4]. In addition, *tet*(M), *tet*(O), *tet*(Q), *tet*(W), and *tet*(32) are coding for ribosomal protection proteins associated with tetracycline resistance in anaerobic bacteria and confer resistance to tetracycline, doxycycline, and minocycline [4,24,111,112,113]. Finally, *tet*(X) is associated with enzymatic degradation [114]. Tigecycline escapes most of the mechanisms of resistance to first- and second-generation tetracyclines due to its slightly different conformation. Few strains with elevated MICs have been identified among anaerobes, and resistant mechanisms still remain unclear [4,115,116].

## 8. Other Agents

Other antibiotics appeared to be active in vitro against anaerobic bacteria, although they are rarely tested routinely and more data on their in vitro activity and clinical effectiveness are needed. Linezolid and tedizolid are oxazolidinone antibiotics, mainly active on Gram-positive bacteria. Studies showed that linezolid had good activity against some Gram-negative anaerobes, including *Bacteroides* spp., *Prevotella* spp., *Fusobacterium nucleatum,* and Gram-positive anaerobes [29,92,117,118,119]. A clinical report confirmed linezolid’s clinical effectiveness in treating anaerobic infections, but there is a lack of published clinical data [120]. Recently, linezolid-resistant isolates of *B. fragilis* (MIC = 8 mg/L) and *C. perfringens* (MIC = 16 mg/L) were identified from zoonotic samples. Genomic analysis reveals the presence of *cfr*(C), previously described in *C. difficile* encoding Cfr(C), a 23S ribosomal methylase linked to cross-resistance to phenicols, lincosamides, oxazolidinones, pleuromutilins, and streptogramins A (the so-called PhLOPS_A_ resistance phenotype) [121]. In *B. fragilis*, *cfr*(C) was located on Tn*6994* in combination with other resistance genes (*nimL*, *ant*(9), *ant*(6)- 1a, *erm*(G), and *tet*(Q)), which may lead to the diffusion of multi-drug resistance across anaerobes [122,123].

Studies testing vancomycin showed that vancomycin had good activity against Gram-positive anaerobes. No resistance was described among Gram-positive cocci with low MIC [18,29,31,68]. Gram-positive bacilli also appeared susceptible to vancomycin. Koizumi et al. and Parisio et al. described no resistant *Cutibacterium* spp. strains, while recent Italian studies reported a low resistance rate (nearly 5%) in *Clostridium* spp. However, as isolates were not identified at the species level, this resistance rate could be influenced by species intrinsically resistant to vancomycin, such as *C. ramosum* or *C. innocuum*. Additionally, Steininger et al. showed that vancomycin is also effective in vitro against *Actinomyces* spp. [90,92,124].

## 9. Discussion

As anaerobic infections are often polymicrobial and mixed with aerobic bacteria, detection, identification, and AST of anaerobic bacteria are cumbersome and not systematically performed in routine microbiological laboratories. However, these bacteria are fully accepted as major pathogens associated with significant morbidity and mortality, especially as their resistance to antibiotics is increasing worldwide. Interpreting and comparing studies becomes challenging due to the difficulties associated with implementing the AST methods recommended by international guidelines. Additionally, the heterogeneity in breakpoint values used for AST interpretation between European and American recommendations further complicates the process. Despite this, global antibiotic resistance surveillance studies have been increasingly published over the past 20 years [11,83]. In all studies, authors observed an alarming increase in antibiotic resistance, and antibiotics that were once considered first-line treatments, such as clindamycin or metronidazole, appear less and less effective. Newer antibiotics, such as tigecycline and linezolid, seemed to be effective against anaerobes with no or low resistance levels. However, more published data are needed to complete our knowledge about them. Our current understanding of the resistance mechanisms that mediate antibiotic resistance in anaerobes is limited, with the majority of studies focusing primarily on *Bacteroides* spp. As a result, there is a need to gather comprehensive data on resistance mechanisms in other anaerobic bacteria to enhance our understanding of this phenomenon. In addition, several studies have shown that the presence of resistance genes is not systematically correlated with phenotypic resistance, and conversely, some susceptible strains harbored resistance genes. This suggests that resistance in anaerobes is complex and that further studies are needed. Worryingly, multidrug-resistant anaerobes have been described in many bacterial species, especially the *B. fragilis* group, with strains that simultaneously harbored a large number of resistance genes, some of which encode multidrug efflux pumps [125,126,127]. In the future, the therapeutic management of these infections may become challenging.

## 10. Conclusions

In conclusion, antibiotic resistance in anaerobes is increasing worldwide, and AST should be performed as far as possible to avoid therapeutic failure. An increasing volume of epidemiological and mechanistic data is being published, highlighting the importance of ongoing laboratory studies on anaerobes. Such research is crucial to fully understanding and preventing resistance in these bacteria.

## Figures and Tables

**Table 1:** MIC values of amoxicillin/clavulanate for Gram-negative anaerobes.

	Method	N	Range MIC	MIC_50_	MIC_90_	References
*Bacteroides fragilis*	E-test	111	0.016–256	0.19	1	[15]
	E-test	324	0.016–256	0.125	2	[20]
	E-test	92	0.125–>256	1	8	[17]
	E-test	63	0.064–>256	2	32	[19]
	E-test	69	0.25–8	0.5	4	[13]
*Bacteroides* spp./ P*arabacteroides* spp.	E-test	111	0.032–32	1	8	[13]
	E-test	38	0.016–256	2	32	[20]
*Fusobacterium* spp.	E-test	21	0.016–4	0.064	1	[13]
	E-test	30	0.016–>256	0.047	0.5	[17]
	E-test	22	≤0.016–2	0.064	0.25	[19]
*Prevotella* spp.	E-test	62	0.016–4	0.38	2	[17]
	E-test	39	≤0.016–32	1	4	[19]
	E-test	45	0.016–2	0.125	1	[13]
*Veillonella* spp.	E-test	17	0.016–8	0.38	8	[17]
	E-test	8	<0.016–2	0.5	2	[13]

**Table 2:** MIC values of penicillin for Gram-positive anaerobes.

	Method	N	Range MIC	MIC_50_	MIC_90_	References
*Actinomyces* spp.	E-test	549	0.002–4	0.06	0.5	[22]
	Agar dilution	23	≤0.06–0.5	0.12	0.12	[21]
*Anaerococcus* spp.	E-test	117	0.002–16	0.12	0.5	[22]
	E-test	26	≤ 0.02–1	0.03	0.25	[28]
*A. prevotii*	E-test	31	0.004–0.25	0.023	0.125	[31]
*Clostridium* spp.	E-test	19	≤0.016–>256	0.25	>256	[19]
	E-test	37	≤0.016–>32	0.094	12	[31]
	E-test	505	≤0.002–64	0.25	2	[22]
	Agar dilution	27	≤0.06–2	0.5	2	[21]
*C. perfringens*	E-test	20	≤0.016–32	0.032	0.064	[19]
	E-test	20	0.016–1.5	0.064	0.25	[31]
	E-test	52	0.03–0.25	0.12	0.12	[28]
	E-test	163	0.0075–64	0.12	0.25	[22]
*Cutibacterium* spp.	E-test	657	0.002–0.5	0.03	0.12	[22]
*C. acnes*	E-test	74	≤0.016–0.064	≤0.016	0.032	[19]
	E-test	40	≤0.016–0.5	0.032	0.094	[31]
*Finegoldia magna*	E-test	31	0.06–0.25	0.12	0.25	[28]
	E-test	37	0.008–0.38	0.125	0.25	[31]
	E-test	32	≤0.016–1	0.064	0.125	[19]
	Agar dilution	31	≤0.06–0.12	≤0.06	≤0.06	[21]
	E-test	120	0.015–0.5	0.12	0.25	[22]
*Eggerthella* spp.	E-test	187	0.004–16	1	4	[22]
	E-test/MIC gradient strip	100	0.06–8	1	2	[32]
*Parvimonas* spp.	E-test	11	≤0.016–0.25	0.016	0.125	[31]
	E-test	40	≤0.002–0.12	0.0075	0.03	[28]
	Agar dilution	29	≤0.06–0.25	0.12	0.25	[21]
	E-test	191	0.002–0.5	0.0075	0.06	[22]
*Peptoniphilus* spp.	E-test	21	0.004–0.25	0.032	0.19	[31]
	E-test	16	≤0.016–1	0.25	0.5	[19]
	E-test	138	0.002–0.5	0.0075	0.06	[22]
*Peptostreptococcus anaerobius*	E-test	19	0.003–2	0.064	0.25	[31]

**Table 3:** MIC values of metronidazole for Gram-negative anaerobes.

	Method	N	Range MIC	MIC_50_	MIC_90_	References
*Bacteroides fragilis*	Agar dilution	60	0.25–8	4	4	[21]
	E-test	111	0.094–0.47	0.023	0.19	[15]
	Microdilution	42	≤2–8	≤2	8	[23]
	E-test	92	0.125–>256	0.75	2	[17]
	E-test	63	0.064–>256	0.125	0.5	[19]
	E-test	485	0.015–>256	1	4	[22]
*Bacteroides fragilis*	Agar dilution	54	0.5–8	2	4	[21]
group	E-test	65	0.064–>256	1	6	[17]
(without *B. fragilis*)	E-test	59	≤0.016–>256	0.125	1	[19]
	E-test	401	0.03–>256	1	4	[22]
*Bacteroides* spp./	Agar dilution	10	1–4	2	4	[21]
*Parabacteroides* spp.						
*Fusobacterium* spp.	Agar dilution	19	0.12–1	≤0.06	1	[21]
	Microdilution	14	≤2	≤2	≤2	[23]
	E-test	34	0.016–≥256	0.032	0.125	[68]
	E-test	30	<0.016–8	0.023	0.5	[17]
	E-test	22	≤0.016–4	≤0.016	0.5	[19]
	E-test	101	≤0.015–4	0.06	0.5	[22]
*Prevotella* spp.	Agar dilution	25	0.125–>8	0.5	8	[26]
	E-test	160	0.016–≥256	0.064	0.5	[68]
	Agar dilution	33	0.12–32	1	8	[21]
	E-test	62	0.016–>256	0.38	4	[17]
	Microdilution	29	≤2–8	≤2	4	[23]
	E-test	39	≤0.016–1	0.125	0.5	[19]
	E-test	244	≤0.015–>256	0.5	2	[22]
*Veillonella* spp.	Agar dilution	11	2–32	8	32	[21]
	E-test	33	0.032–≥256	1	4	[68]
	E-test	17	0.023–8	0.75	3	[17]
	E-test	73	≤0.016–>256	4	8	[22]
*V. parvula*	E-test	14	0.064–8	1	8	[19]

**Table 4:** MIC values of metronidazole for Gram-positive anaerobes.

	Method	N	Range MIC	MIC_50_	MIC_90_	References
*Actinomyces* spp.	E-test	549	0.03–≥512	512	512	[22]
	Agar dilution	23	32–>128	>128	>128	[21]
*Anaerococcus* spp.	Agar dilution	10	0.25–2	1	2	[29]
	E-test	117	≤0.015–16	0.50	2	[22]
*A. prevotii*	E-test	31	0.023–>256	0.25	>256	[31]
*Clostridium* spp.	E-test	71	0.016–≥256	0.5	4	[68]
	E-test	19	≤0.016–1	0.064	0.5	[19]
	E-test	37	0.16–>256	0.125	4	[31]
	E-test	504	≤0.015–8	0.5	4	[22]
	Agar dilution	27	0.25–64	2	8	[21]
*C. perfringens*	E-test	20	0.25–32	1	4	[19]
	E-test	20	0.5–6	1.5	2	[31]
	E-test	163	0.25–32	2	8	[22]
*Cutibacterium* spp.	E-test	657	0.25–>512	512	512	[22]
*C. acnes*	E-test	40	>256	>256	>256	[31]
*Finegoldia magna*	Agar dilution	49	0.25–4	0.5	1	[29]
	E-test	37	0.064–>256	0.38	2	[31]
	E-test	32	≤0.016–1	0.125	1	[19]
	Agar dilution	31	0.12–8	1	1	[21]
	E-test	100	0.016–≥256	0.125	256	[68]
	E-test	120	≤0.015–4	0.5	2	[22]
*Eggerthella* spp.	E-test	187	≤0.015–512	0.5	4	[22]
	Agar dilution	38	0.5–1	1	1	[21]
*Parvimonas* spp.	Agar dilution	33	0.12–4	0.25	0.5	[29]
	E-test	11	0.032–>256	0.25	0.75	[31]
	E-test	17	≤0.016–>256	0.064	2	[19]
	Agar dilution	29	0.5–4	1	2	[21]
	E-test	39	0.016–≥256	0.032	0.25	[68]
	E-test	191	≤0.015–8	0.25	1	[22]
*Peptoniphilus* spp.	Agar dilution	30	0.12–4	1	2	[29]
	E-test	21	0.064–>256	0.25	16	[31]
	E-test	16	≤0.016–>256	0.5	1	[19]
	E-test	138	≤0.015–8	0.25	2.6	[22]
*Peptostreptococcus*	Agar dilution	11	0.25–1	0.5	1	[29]
*anaerobius*	E-test	19	0.023–0.5	0.125	0.38	[31]

**Table 5:** MIC values of clindamycin for Gram-negative anaerobes.

	Method	N	Range MIC	MIC_50_	MIC_90_	References
*Bacteroides fragilis*	Agar dilution	60	≤0.06–>128	1	>128	[21]
	E-test	111	0.016–256	0.5	256	[15]
	Microdilution	42	0.5–32	1	>16	[23]
	E-test	92	0.032–>256	1.5	>256	[17]
	E-test	63	≤0.016–>256	0.5	>256	[19]
	E-test	472	≤0.015–>512	2	512	[22]
*Bacteroides fragilis*	Agar dilution	54	≤0.06–>128	>128	>128	[21]
Group	Microdilution	37	0.5–32	>16	>16	[23]
(without *B. fragilis*)	E-test	65	0.064–>256	>256	>256	[17]
	E-test	59	≤0.016–>256	8	>256	[19]
	E-test	392	≤0.015–>512	8	512	[22]
*Bacteroides*/	Agar dilution	10	0.5–128	>128	>128	[21]
*Parabacteroides* spp.						
*Fusobacterium* spp.	Agar dilution	19	≤0.06–>128	2	16	[21]
	Microdilution	14	0.25–16	≤0.5	4	[23]
	E-test	34	0.016–≥256	0.032	256	[68]
	E-test	30	0.016–≥256	0.047	0.38	[17]
	E-test	63	≤0.016–>256	0.047	0.38	[19]
	E-test	97	≤0.015–>512	0.03	2	[22]
*Prevotella* spp.	Agar dilution	33	≤0.06–>128	≤0.06	>128	[21]
	E-test	62	<0.016–>256	0.047	>256	[17]
	E-test	160	0.008–≥256	0.032	256	[68]
	Microdilution	29	0.5–32	>16	>16	[23]
	E-test	39	≤0.016–>256	0.064	>256	[19]
	E-test	241	≤0.015–>512	64	512	[22]
*Veillonella* spp.	Agar dilution	11	≤0.06–>128	≤0.06	2	[21]
	E-test	33	0.016–≥256	0.125	0.5	[68]
	E-test	17	0.016–>256	0.125	>256	[17]
	E-test	14	≤0.016–>256	0.064	0.25	[19]
*V. parvula*	E-test	73	≤0.015–>512	0.12	0.9	[22]

**Table 6:** MIC values of clindamycin for Gram-positive anaerobes.

	Method	N	Range MIC	MIC_50_	MIC_90_	References
*Actinomyces* spp.	E-test	542	≤0.015–>512	0.25	512	[22]
	Agar dilution	23	≤0.06–>128	0.25	>128	[21]
*Anaerococcus* spp.	Agar dilution	10	≤0.03–>32	0.06	16	[29]
	E-test	114	≤0.015–>512	0.12	512	[22]
*A. prevotii*	E-test	31	0.016–>256	0.25	>256	[31]
*Clostridium* spp.	E-test	71	0.016–>256	2	16	[68]
	E-test	19	≤0.016–>256	0.25	32	[19]
	E-test	37	0.016–>256	0.25	>256	[31]
	E-test	491	<0.015–>512	1	16	[22]
	Agar dilution	27	≤0.06–>128	1	>128	[21]
*C. perfringens*	E-test	20	≤0.016–>256	2	4	[19]
	E-test	20	0.032–>256	2	>256	[31]
	E-test	29	0.032–8	2	4	[68]
	E-test	160	0.03–>512	2	4	[22]
*Cutibacterium* spp.	E-test	637	≤0.015–>512	0.06	5.6	[22]
*C. acnes*	E-test	74	≤0.016–>256	≤0.016	0.125	[19]
	E-test	40	0.023->256	0.094	1	[31]
*Finegoldia magna*	Agar dilution	49	≤0.03–>32	1	32	[29]
	E-test	37	0.032–>256	6	>256	[31]
	E-test	32	0.032–>256	0.5	>256	[19]
	Agar dilution	31	≤0.06–64	≤0.06	0.5	[21]
	E-test	100	0.016–>256	0.5	256	[68]
	E-test	115	≤0.015–>512	2	512	[22]
*Eggerthella* spp.	E-test	180	≤0.015–>512	0.25	1	[22]
*Parvimonas* spp.	Agar dilution	33	≤0.03–>32	0.12	1	[29]
	E-test	11	0.047–>256	0.125	16	[31]
	E-test	17	≤0.016–32	0.064	0.125	[19]
	Agar dilution	29	≤0.06–128	1	128	[21]
	E-test	39	0.016–>256	0.125	256	[68]
	E-test	188	≤0.015–>512	0.25	1	[22]
*Peptoniphilus* spp.	Agar dilution	30	≤0.03–>32	1	>32	[29]
	E-test	21	0.016–>256	1.5	>256	[31]
	E-test	16	≤0.032–>256	0.064	>256	[19]
	E-test	136	≤0.015–>512	0.25	512	[22]
*Peptostreptococcus*	Agar dilution	11	≤0.03–>32	0.5	1	[29]
*anaerobius*	E-test	19	0.023–>256	0.38	>256	[31]

**Table 7:** Mechanisms of resistance in anaerobes.

Antibiotic	Resistance Mechanism	Antibiotic Resistance Element	Example of Species
**Β-lactams**	Β-lactamase		
	Penicillinase		*Fusobacterium* spp., *Clostridium* spp, *Porphyromonas* spp.
	Cephalosporinase	CepA	*B. fragilis* group
		CfxA	*B. fragilis* group, *Prevotella* spp.
	Carbapenemase	CfiA	*B. fragilis* group
	PBP-alteration	PBP1, PBP2, PBP3, PBP2Bfr	*B. fragilis* group
		PBP1, PBP6	*Veillonella* spp.
	Reduce uptake of drug	BmeABC	*B. fragilis* group
	Loss of porin channels		*B. fragilis*
**Metronidazole**	Drug inactivation	NimA-H, Nim J	*Bacteroides* spp
		NimA–C, NimI, Nim K	*Prevotella* spp.
		NimB	*Peptostreptococcus* spp., *F. magna*, *A. prevotti*, *P. micra*
		NimC	*Porphyromonas* spp.
		NimD	*Fusobacterium* spp.
		NimE	*Veillonella* spp.
	Metabolic changes		*B. fragilis* group
	Reduce uptake of drug	BmeABC	*B. fragilis* group
	Increase DNA repair	RecA	*B. fragilis*
**Clindamycin**	rRNA methylases	ErmB, ErmF and ErmG	*B. fragilis* group, *Prevotella* spp.
		ErmX	*Cutibacterium* spp.
	Reduce uptake of drug	MsrSA and MefA	*B. fragilis* group
**Fluoroquinolones**	Target modification	GyrA and ParC	*B. fragilis* group
	Reduce uptake of drug	BexA	*B. fragilis* group
**Chloramphenicol**	Drug inactivation	Cat	*B. fragilis* group
**Tetracyclines**	Reduce uptake of drug	TetA–E	*B. fragilis* group
		TetK and TetL	*Peptostreptococcus* spp., *Veillonella* spp.
	Drug inactivation	TetX	*B. fragilis* group
	Ribosomal protection	TetM and TetQ,	*B. fragilis* group, *Peptostreptococcus* spp., *Clostridium* spp., *Prevotella* spp., *Fusobacterium* spp.
		TetW	*Veillonella* spp., *Prevotella* spp.
		Tet32	*Clostridium* spp.,
**Linezolid**	Ribosomal protection	Cfr(C)	*B. fragilis*, *C. perfringens* (animal isolates)

## Data Availability

No new data were created or analyzed in this study. Data sharing is not applicable to this article.
